# Content Analysis of Negative Online Reviews of Hospice Agencies in the United States

**DOI:** 10.1001/jamanetworkopen.2019.21130

**Published:** 2020-02-05

**Authors:** Elinor J. Brereton, Daniel D. Matlock, Monica Fitzgerald, Grace Venechuk, Chris Knoepke, Larry A. Allen, Channing E. Tate

**Affiliations:** Adult and Child Consortium for Outcomes Research and Delivery Science, Anschutz Medical Campus, University of Colorado School of Medicine, Aurora; Adult and Child Consortium for Outcomes Research and Delivery Science, Anschutz Medical Campus, University of Colorado School of Medicine, Aurora; Division of Geriatrics, University of Colorado School of Medicine, Aurora; VA Eastern Colorado Geriatric Research Education and Clinical Center, Denver; Adult and Child Consortium for Outcomes Research and Delivery Science, Anschutz Medical Campus, University of Colorado School of Medicine, Aurora; Adult and Child Consortium for Outcomes Research and Delivery Science, Anschutz Medical Campus, University of Colorado School of Medicine, Aurora; Adult and Child Consortium for Outcomes Research and Delivery Science, Anschutz Medical Campus, University of Colorado School of Medicine, Aurora; Division of Cardiology, University of Colorado School of Medicine, Aurora; Adult and Child Consortium for Outcomes Research and Delivery Science, Anschutz Medical Campus, University of Colorado School of Medicine, Aurora; Division of Cardiology, University of Colorado School of Medicine, Aurora; Adult and Child Consortium for Outcomes Research and Delivery Science, Anschutz Medical Campus, University of Colorado School of Medicine, Aurora

## Abstract

**IMPORTANCE:**

As online reviews of health care become increasingly integral to patient decision-making, understanding their content can help health care practices identify and address patient concerns.

**OBJECTIVE:**

To identify the most frequently cited complaints in negative (ie, 1-star) online reviews of hospice agencies across the United States.

**DESIGN, SETTING, AND PARTICIPANTS:**

This qualitative study conducted a thematic analysis of online reviews of US hospice agencies posted between August 2011 and July 2019. The sample was selected from a Hospice Analytics database. For each state, 1 for-profit (n = 50) and 1 nonprofit (n = 50) hospice agency were randomly selected from the category of extra-large hospice agencies (ie, serving >200 patients/d) in the database. Data analysis was conducted from January 2019 to April 2019.

**MAIN OUTCOMES AND MEASURES:**

Reviews were analyzed to identify the most prevalent concerns expressed by reviewers.

**RESULTS:**

Of 100 hospice agencies in the study sample, 67 (67.0%) had 1-star reviews; 33 (49.3%) were for-profit facilities and 34 (50.7%) were nonprofit facilities. Of 137 unique reviews, 68 (49.6%) were for for-profit facilities and 69 (50.4%) were for nonprofit facilities. A total of 5 themes emerged during the coding and analytic process, as follows: discordant expectations, suboptimal communication, quality of care, misperceptions about the role of hospice, and the meaning of a good death. The first 3 themes were categorized as actionable criticisms, which are variables hospice organizations could change. The remaining 2 themes were categorized as unactionable criticisms, which are factors that would require larger systematic changes to address. For both for-profit and nonprofit hospice agencies, quality of care was the most frequently commented-on theme (117 of 212 comments [55.2%]). For-profit hospice agencies received more communication-related comments overall (34 of 130 [26.2%] vs 9 of 82 [11.0%]), while nonprofit hospice agencies received more comments about the role of hospice (23 of 33 [69.7%] vs 19 of 31 [61.3%]) and the quality of death (16 [48.5%] vs 12 [38.7%]).

**CONCLUSIONS AND RELEVANCE:**

Regarding actionable criticisms, hospice agencies could examine their current practices, given that reviewers described these issues as negatively affecting the already difficult experience of losing a loved one. The findings indicated that patients and their families, friends, and caregivers require in-depth instruction and guidance on what they can expect from hospice staff, hospice services, and the dying process. Several criticisms identified in this study may be mitigated through operationalized, explicit conversations about these topics during hospice enrollment.

## Introduction

Hospice is a consistently underused arm of the health care sector.^1–3^ This is thought to be caused in part by negative stigmas surrounding death, with hospice perceived as its harbinger, as well as misperceptions about affordability and eligibility.^4,5^ However, this sentiment is incongruent with studies showing that hospice care often improves quality of life for both patients and loved ones and, in some cases, even extends life when compared with patients seeking active treatments.^6-8^ As work to mitigate negative stigmas regarding hospice and to improve use continues, online reviews can elucidate public perceptions of hospice, reflecting concerns regarding the actual care received and a broader cultural aversion to death and the dying process. These reviews are also resources for the general public, who often use them to make decisions about their health care. In a 2017 study of online researching habits for individuals seeking hospice,^9^ our group found that, when asked to conduct a web search for information about hospice, 55% of participants visited an online review site; half of them visited Yelp.com specifically. Yelp.com is an online platform that allows consumers to rate and review businesses and services. All comments and reviews are made public and are easily accessible on the website. Reviews are scored on a 1-to 5-star rating scale, with 1 star indicating the lowest rating and 5 stars indicating the highest rating.

As of 2016, the number of Yelp reviews evaluating health care experiences in the United States averaged 42 000 per month.^10^ These reviews spanned the health sector and included comments from former employees, caregivers, and patients. Although there are concerns that these reviews do not accurately reflect the quality of care provided,^11,12^ other studies have shown a strong association between online reviews of health care facilities and the scores they receive from formal surveys of patient satisfaction as well as measures of technical quality of care.^6,9-15^ These associations as well as Yelp’s fake-review filter^16,17^ have made the evaluation of Yelp scores a legitimate focus of health research. Because of the increasing role of online reviews in health care decision-making, it is important to understand how these reviews can be used by health care providers, such as hospice agencies.

Thus far, most research on online reviews has focused on the star rating a facility receives. Our study expands on this approach by analyzing the content of 1-star reviews of hospice agencies across the United States. We specifically chose 1-star reviews because they have the potential to be overly influential on readers’ opinions. Additionally, exploring the words of the most critical respondents might best elucidate barriers to hospice use. The purpose of this study was to identify the themes that are consistently cited in the most negative online reviews of diverse hospice agencies across the United States.

## Methods

### Study Design

We conducted a qualitative thematic analysis of 1-star online reviews of US hospice agencies posted on Yelp.com between August 2011 and July 2019. The institutional review board at the University of Colorado approved this study as exempt, granting the study a waiver of documentation of informed consent because of the minimal risk associated with this study as well as the deidentification of any hospice agencies or reviewers. The study followed the Consolidated Criteria for Reporting Qualitative Research (COREQ) reporting guideline to the greatest extent possible, omitting only those areas that directly address forms of data gathering not used (eg, interviews, focus groups) or methods that were not possible because of ethical considerations (eg, member checking).

### Data Collection and Sample

The sample was selected from the online InfoMAX database (Hospice Analytics).^18^ This database categorizes hospice agencies by location, size, status (ie, for profit or nonprofit), and cost. The 2 categories of importance for this sample were status and size. For size, hospice agencies are categorized by how many patients are served daily, with the largest category (ie, extra large) serving more than 200 patients per day. The team used this category for our sample because we believed it would yield the greatest number of Yelp reviews owing to the sheer volume of people involved in these hospice agencies’ services. We used a random number generating website^15^ to produce numbers that also appeared on the numerical list of the largest for-profit and nonprofit hospice agencies in the analytic database (ie, if the number-generating website produced the number 7, then the agency listed as number 7 on the online database was selected for the sample). For each US state, 1 for-profit (n = 50) and 1 nonprofit (n = 50) hospice agency were selected from the extra-large category in the database (N = 100). Yelp.com was then searched for each selected hospice agency. If the selected hospice agency did not have a 1-star reviews, the research team sequentially went down the numerical list of hospice agencies from the same state and with the same status in the database until a hospice agency with a 1-star review was identified.

### Statistical Analysis

Reviews were coded by a trained qualitative researcher (E.J.B.) in Atlas.ti version 8 (Atlas.ti) using a codebook created inductively by members of the research team (E.J.B., D.D.M., and C.E.T.), which included researchers and clinicians with expertise in end-of-life care, disparities in hospice use, and qualitative research. Codes were selected based on their frequency in the data and their relevance to the research question.^19^ Once codes were selected, the research team organized codes into themes based on shared subject matter. The themes were categorized into 2 domains using a thematic analysis approach by structuring the results in a cohesive and meaningful manner based on established qualitative methods and the interpretation of the research team.^20^ The codebook was cross-referenced by the multidisciplinary research team (E.J.B., D.D.M., and C.E.T.) and reapplied to the data to ensure reliability. The research team reviewed the results of the study at several intervals during the analytic process to ensure credibility of the results. The descriptive statistics comparing for-profit and nonprofit hospice agencies were compiled in Excel Office 365 (Microsoft Corp).

## Results

Of the 50 US states, 17 did not have any for-profit hospice agencies with a 1-star review, and 16 did not have any nonprofit hospice agencies with a 1-star review, leaving 67 hospice agencies in our sample. Of these, 33 (49.3%) were for-profit hospice agencies, and 34 (50.7%) were nonprofit. They had a total of 137 unique 1-star reviews. While the number of 1-star reviews only differed by 1 between for-profit (n = 68 [49.6%]) and nonprofit (n = 69 [50.4%]) hospice agencies, 1-star reviews accounted for 68 of 123 total reviews (55.3%) of for-profit hospice agencies and 69 of 202 total reviews (34.2%) of nonprofit hospice agencies in the sample (Table 1).

The range of criticisms was relatively narrow, allowing the research team to easily identify shared areas of concern across the reviews. We identified 5 major themes, categorized into 2 domains. While the research team acknowledges that these domains and themes overlap (eg, suboptimal communication jeopardizes the quality of care), these categorizations were applied to isolate criticisms for thorough analysis.

### Actionable Criticisms

This domain included criticisms that appeared to fall under the purview, control, and responsibility of individual hospice agencies. This domain encompassed 3 themes, as follows: discordant expectations, suboptimal communication, and the quality of care.

### Discordant Expectations

The theme of discordant expectations was defined as reviews that conveyed a disconnect between what a reviewer was explicitly told their experience of hospice care would be and what they actually experienced. Reviewers often used different iterations of the phrase “promises were made but not kept” to describe their disappointment and surprise. This phraseology was used in the context of eligibility and discharge criteria, education, and family support. Reviewers discussed not understanding eligibility criteria, having eligibility criteria change while their loved one was receiving care, and having loved ones discharged from hospice unexpectedly. They described not receiving the education they were told they would receive, that they expected, or that they felt was needed to administer care and medications to loved ones at home, and they felt less family support from staff than they had anticipated (Table 2).

### Suboptimal Communication

Suboptimal communication was closely related to discordant expectations. Reviewers described their experience of being approached by hospice enrollment staff as feeling more like being given a sales pitch than working with someone who had a genuine interest in providing care and comfort to their family members and loved ones, and some felt rushed and pressured to make decisions about enrollment. Once enrolled, reviewers described a lack of internal communication among staff members, which negatively affected the cohesiveness and quality of their loved ones’ care. However, the most frequently identified communication concern was the inability to reach staff by telephone, especially during emergencies, and the distress this caused those who were providing care for their loved ones, especially at home (Table 2).

### Quality of Care

Quality-of-care concerns were defined as criticisms pertaining to the day-to-day logistical tasks of hospice care. These concerns accounted for the largest category of comments (117 of 212 comments [55.2%]) in all 1-star reviews from both for-profit and nonprofit hospice agencies. We found frequent mentions of visitation issues, centered on the frustration of not knowing when, or even if, staff would arrive for their anticipated appointments. Reviewers wrote about the quality of the staff members themselves and their ability to perform the necessary tasks associated with hospice care. Some reviewers blamed management for these issues, arguing that staff were too overworked or understaffed to provide adequate care. Reviewers also wrote about the frequency of staff turnover, issues with broken or absent equipment, inadequate wound management, and issues regarding medication (Table 2).

### Unactionable Criticisms

The second domain included criticisms associated with hospice care and the dying process that fell outside the scope of an individual hospice agency or its staff. While the themes in the first domain could be acted on by individual hospice organizations, this domain encompassed themes that spoke to a greater cultural aversion to and misunderstanding of death and the dying process. This domain was divided in 2 themes, as follows: misperceptions about the role of hospice and the meaning of a good death.

### Misperceptions About the Role of Hospice

The research team selected quotes for this theme that described reviewers’ beliefs about the role of hospice care, what the mission and philosophy of hospice care is, and what motivates hospice agencies and staff. Many of these comments were directed to larger systemic and philosophical issues that lie outside the control and scope of individual hospice agencies and staff members to address and may instead reflect a lack of exposure to or education on hospice before the need to enroll a loved one arose. In contrast to the theme of discordant expectations, these comments were not made in response to information given to reviewers by staff or individual agencies. Instead, reviewers wrote about the things they thought hospice was supposed to be and do and how they believed the experience fell short of these goals, including by not upholding the mission of hospice, having motivations at odds with these goals, and hastening death. Reviewers often connected the hospice agency’s inability to achieve these purported goals by identifying conflicting interests, such as financial gain (Table 3).

### The Meaning of a Good Death

Many reviewers wrote about the suffering of their loved ones while receiving hospice care and the inability of staff to adequately comfort patients, especially during their final moments. Reviewers shared the indignities and pain their loved ones experienced and the absence of guidance, support, and empathy from staff. These comments reflected a conflict that has been noted in the literature, ie, that hospice is selected to provide patients with a good death but that this ideal is often unachievable because of a myriad of factors outside an individual hospice agency’s or staff member’s control. The most common hopes expressed by reviewers were the ideals of peacefulness or comfort, dignity, preparedness, and awareness. Additionally, reviewers expressed a strong desire to have the death of their loved ones have meaning, accomplished through shared witnessing or grief with hospice staff. Reviewers described conflicting perceptions of death’s meaning in comments describing staff not being present at the moment of death and providing impersonal follow-up and condolences after death (Table 3).

### Comparison of For-Profit With Nonprofit Hospice Agencies

The most significant difference found between hospice types in this sample was that for-profit hospice agencies received 5.7-fold more negative comments about telephone communication issues than nonprofit hospice agencies (23 of 130 [17.7%] vs 4 of 82 [4.9%]) (Figure). For-profit hospice agencies received 4.3-fold more comments about promises being made but not kept (17 [13.1%] vs 4 [4.9%]) and 4.5-fold more comments about equipment-related problems (9 [6.9%] vs 2 [2.4%]) (Figure). Nonprofit hospice agencies received 3.8-fold more comments about confusion regarding eligibility or discharge (15 [18.3%] vs 4 [3.1%]) (Figure). For both for-profit and nonprofit hospice agencies, quality of care was the most frequently commented-on theme (117 of 212 comments [55.2%]). For-profit hospice agencies received more communication-related comments overall (34 of 130 [26.2%] vs 9 of 82 [11.0%]), while nonprofit hospice agencies received more comments about the role of hospice (23 of 33 [69.7%] vs 19 of 31 [61.3%]) and the quality of death (16 [48.5%] vs 12 [38.7%]).

## Discussion

The 1-star reviews from this sample showed that the quality of care received while in hospice was the most frequent concern for reviewers. In addition, there were important concerns regarding false promises; this group of negative reviews, while less frequent, indicated that hospice care and what it can offer, including the ability to always provide a good death, may have been oversold to reviewers. These findings can be organized in 2 major domains: complaints that may be actionable by individual hospice agencies and those that are not. Complaints identified as actionable were consistent with previous research on both formal and informal patient complaints regarding health care and hospice. Complaints identified as unactionable appear consistent with sociological research on the emblematic nature of hospice and its role in achieving a personally and culturally defined good death.

A review of hospice care complaints placed through the Centers for Medicare & Medical Services from 2005 to 2015 listed the most frequently cited complaints as follows: (1) the quality of care (45%), (2) patients’ rights violations during admission and/or discharge (20%), and (3) administrative and/or personnel concerns (14%).^21^ These categories are consistent with the Patient Complaint Taxonomy,^11^ which sampled patient complaints from PubMed, Science Direct, and Medline databases and categorized the findings into the 3 following domains: clinical (34%), management (35%), and relationships (29%). These echo categories of complaints sampled from Web of Science, Scopus, and PubMed, which found the most frequent categories of complaint to be patient-centered processes (50%), appropriate knowledge and skills of physicians (25%), and the care environment (24%).^22^ Although the themes used for this study differ slightly from these examples, there is substantial overlap in complaint categories, illustrating concerns about the quality of care patients receive, issues with expectations regarding eligibility and services developed during admission, issues with communication, and administrative and/or managerial concerns. These similarities also revealed that, although online complaints often do not yield the sort of administrative or remedial action that formal complaints may evoke, they do provide consistent and insightful criticisms that can effectively direct quality improvement initiatives.

Of particular interest to this study, previous research has identified marked inconsistencies among hospice agencies regarding how patients are enrolled and educated during admission.^4^ These inconsistencies have unknown implications for patient outcomes, although the analysis from this study suggests that incomplete, inaccurate, misleading, or impersonal admission processes may factor into the negative feelings and confusion reviewers described in their online reviews. Many comments mentioned that promises were made but not kept, indicating that these initial meetings with hospice enrollment staff provide an opportunity to set realistic expectations about the care and services that can and will be provided for patients and their families, friends, and caregivers.

Of note, the differences between for-profit and nonprofit facilities identified in this study are also consistent with the literature. Our observation that for-profit hospice agencies were more likely to receive a negative review in general is congruent with research conducted in nursing home and hospice settings, which demonstrated that for-profit facilities are of lower quality.^23,24^ For-profit facilities differ from nonprofit hospice agencies in that they are required to pay state and federal taxes and incentivized to reduce costs by lowering the quantity and quality of services provided.^23^

The ability of hospice staff to accomplish information-sharing completely, accurately, and compassionately during initial consultations and enrollments may be inhibited by time limitations and external challenges. In 2016, the national median time patients spent receiving hospice care before death was 17 days, with 27.9% of patients spending fewer than 7 days enrolled.^25^ With patients enrolling so close to death, it may be difficult to have a sufficient discussion of expectations while simultaneously trying to initiate care for patients in need of immediate services. Staff may also experience the added difficulty of presenting information during enrollment that they hope to be accurate but that may later go unfulfilled because of challenges such as staff shortages, miscommunication, and unforeseen complications. Although these barriers are substantial and difficult to navigate, this study identified the continued need to improve and operationalize the enrollment and admissions process to promote effective communication between staff and patients and to establish realistic expectations regarding hospice care, hospice staff, and the dying process.

Complaints that do not appear to be addressable by individual hospice agencies are also consistent with literature exploring the concept of a good death and the role of hospice in accomplishing that goal. A good death, as it is often defined in Western societies, is understood to involve “dignity, peacefulness, preparedness, awareness, adjustment, and acceptance.” ^21^ In this study, critical reviews of hospice agencies expressed shock and distress that the death of their loved one deviated so substantially from the quality of death they anticipated getting through hospice. This can be seen most poignantly in comments in which reviewers compared previous positive experiences with hospice organizations that provided a good death. Hospice agencies that accomplished this goal were described as being able to manage the pain and suffering of patients at the end of life, to help families, friends, and caregivers prepare by giving them an accurate timeline for the patient’s death, to preserve the dignity of patients, and to promote meaning in death through the witnessing and shared grieving of the patient’s death. While all of these attributes may not be possible with a given patient, increased communication between staff and families, friends, and caregivers about the dying process and what hospice can provide may help manage these expectations. In response to many comments about staff turnover and a lack of cohesion in care teams for patients, an increased emphasis on building relationships with patients and their families, friends, and caregivers, helping families, friends, and caregivers engage their own communities during their loved ones’ time in hospice, and providing more cohesive care may also promote feelings of shared meaning and support.

Our findings are consistent with the recent Office of the Inspector General (OIG) report highlighting that more consistent and operationalized oversight of hospice agencies is needed.^26^ This includes more regulations around communication and shared decision-making. Our results confirm that there are major deficiencies in hospice communication and the regulatory environment. Furthermore, the Office of the Inspector General report noted that one-third of hospice agencies have formal registered complaints against them.^26^ Given the increase in number of health care consumers relying on online reviews, it may be prudent for regulatory agencies, such as the Centers for Medicare & Medicaid Services, to pay attention to online reviews when formulating policies.

### Limitations

This study has limitations. Online reviews are unique because they lack the processual barriers found in more formal complaint submission platforms. This informal platform increases access for people wanting to submit reviews and captures their reaction to an event in real-time. While this broadens the theoretic pool of individuals who might submit negative reviews and increases the range of the types of complaints that would be considered appropriate, this lack of processual barriers means that very little data about the reviewer and the context of the review are available to those reading the reviews. This limitation means that reviews must be taken at their content value only.

Another limitation is our relatively small sample size. Give than there are more than 4500 hospice agencies in the United States,^18^ it was not feasible to sample them all because of both the volume and the fact that not all have online reviews. We devised a random national sampling strategy that, although small in size, revealed shared criticisms across the United States and across different types of hospice organizations.

We were also unable to code for demographic information, such as sex, age, socioeconomic status, and race/ethnicity. Focusing on 1-star reviews only allowed for an analysis of the concerns and problems faced by users of hospice; it provided a 1-sided view that limited context and may have overemphasized outliers. Furthermore, this is a limited sample used to identify general themes, and the relative incidence of criticisms should be interpreted with caution.

## Conclusions

Hospice care continues to be underused in the face of data showing improved length and quality of life. By identifying concerns shared online by patients and their families, friends, and caregivers through informal websites, hospice agencies and other health care agencies can become better informed regarding the needs and dissatisfactions of their clients, and medical systems in general can better address cultural and systematic issues that limit the use and benefit of hospice care. For the items categorized as actionable criticisms, hospice agencies could examine their current practices, given that reviewers described these issues as negatively affecting the already difficult situation of losing a loved one. Furthermore, patients and their families, friends, and caregivers require in-depth instruction and guidance on what they can expect from hospice staff, hospice services, and the dying process. Several criticisms identified in this study may be mitigated through operationalized conversations regarding these topics during hospice enrollment. While not all criticisms are actionable by individual agencies, all criticisms do add to the broader picture of what causes families, friends, and caregivers pain and distress during their time with hospice and the death of their loved ones.

## Figures and Tables

**Figure.**
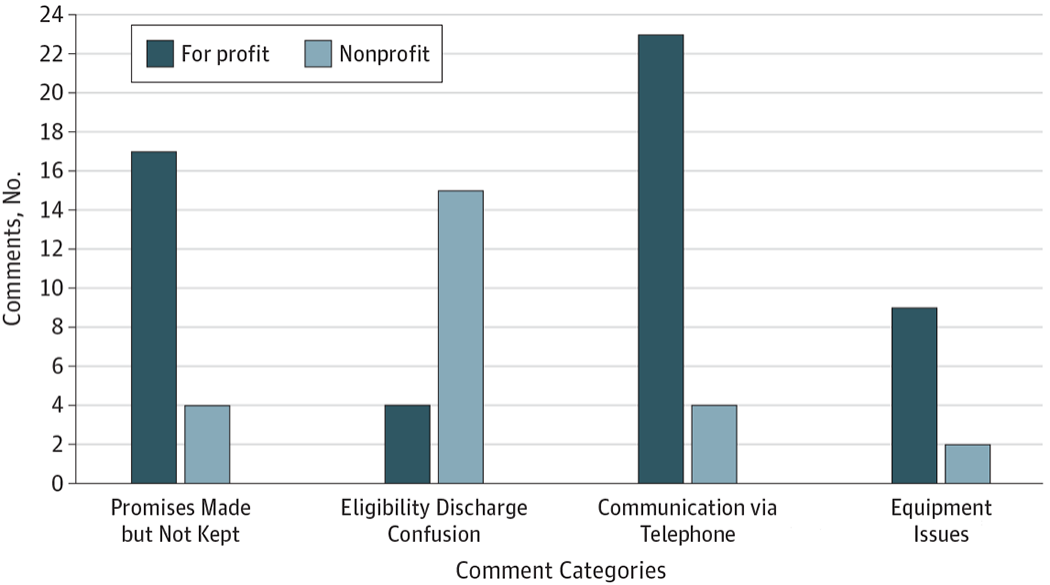
Differences Between Comments Describing For-Profit and Nonprofit Hospice Agencies

**Table 1.** Differences Between Reviews of For-Profit and Nonprofit Hospice Agencies

	No. (%)	
Characteristic	For-Profit Hospice Agencies (n = 33)	Nonprofit Hospice Agencies (n = 34)
All reviews	123 (37.8)	202 (62.2)

1-Star reviews	68 (49.6)	69 (50.4)

Star rating, mean (SD)	2.19 (1.08)	2.50 (1.21)

**Table 2.** Actionable Criticisms, With Themes of Discordant Expectations, Suboptimal Communication, and Quality of Care

Key Themes	Quotes
Discordant expectations	
Promises made but not kept	“It started with all the wonderful promises the ‘sales person’ made. I don’t think she knows her company and what they really provide. They are a business of promises not kept. And there’s no returns. I will never get the last days of my father again.” ^a^
Eligibility or discharge confusion	“We were extremely disappointed when my father was abruptly discharged saying that he improved slightly in his ADLs [activities of daily living] and weight was stable. During the 5 months that my father was in hospice care he declined as expected[,] and I find it hard to believe that the nurses were reporting he had improved.”^b^
Education	“The nurse never answered any question that my mother-in-law had! Patient education should be a top priority, especially if they only get to see a home health nurse once a day/every other day.”^a^
Family support and aftercare	“It has been 11 days, and we are still waiting for the promised grief counseling and chaplain services. My 91 yr old mother-in-law (living alone in assisted living) is a mess, and still no Chaplain. We’re waiting ”^a^
Suboptimal communication	
Sales pitch	“She wanted me to sign immediately, put heavy pressure on me saying if I don’t sign I’m not doing the right thing for my mother[.…] This is at a time when emotional decisions must be made for the best of your loved ones and they prey on people who do not have enough information to make a good decision.”^a^
Internal communication	“Everything was horrible from the beginning, starting with the lack of communication between employees at the hospice.”^a^
Communication via telephone	“We called the night nurse, no answer. We called our contact, no answer. We called all the numbers we had from [hospice name redacted]…no one answered. When we finally got in touch with someone [it] was hours after he ha[d] been dead already.”^a^
Quality of care	
Visitations	“This location is a MESS! They’re never on time, or just don’t show up period.”^b^
Quality of staff	“When at home, the visiting nurses were uncaring and incompetent. I know that it’s a tough job and so hospice would not attract talented and bright staff.”^b^
Management and organization	“Even after he had passed they called us the next day to tell us a nurse was coming later that day to care for him!!! What kind of organization are they running?”^a^
Overworked and understaffed	“There were a few that really did have a heart, but most were overworked and did not take the time for him.”^a^
Staff turnover	“Not only did we NOT receive quality care[,] we never had continuity of care! A different jackass for each day!”^a^
Equipment	“Equipment broken, fixed with packing tape (WTH), unable to get supplies for DAYS and difficult to communicate.”^b^
Medication	“My mother was in such pain, getting the pain under control was frustrating. We had to fight every day for meds for my mother.”^a^

a For-profit hospice agency.

b Nonprofit hospice agency.

**Table 3.** Unactionable Criticisms, With Themes of Misperceptions About the Role of Hospice and the Meaning of a Good Death

Key Themes	Quotes
Misperceptions about the role of hospice	
The mission or philosophy of hospice	“Hospice is suppose[d] to be empathetic, and above all they are suppose[d] to ensure that patients pass over with dignity and there as absolutely no evidence of that with [hospice name redacted].”^a^
Sped-up death	“They ‘forced us’ to go here after being at home and then planned to speed up the death with extra pain meds and Ativan.”^b^
Motivations of hospice agencies and staff	“I am amazed of how much hospice companies vary. They vary in service but more importantly they vary in customer service and sensitivity. […] Some care about people, some care about money.”^a^
Family support	“During the terminal and final stages where you really need someone there, you are on your own.”^a^
Meaning of a good death	
Dignity	“My father died in a nursing home like a dog[. …] Our dogs have been treated with more dignity and respect in their final days[. …] Pain unmanaged and no support through hospice, my experience with hospice and the dying process has been beyond horrible.”^b^
Peacefulness and comfort	“My mother’s last few days were far from comfortable in this facility. I am devastated by the suffering she endured her last few days in this world.”^b^
Preparedness	“Finally, the only [hospice name redacted] nurse I had faith in, came in and confirmed that end was very near. But no time to get my family in.”^a^
Awareness	“For the last 4 days of her life, she was pain free, but unable to speak or communicate. It was pure torture for me to watch.”^b^
Conflicting perceptions of death’s meaning	“They were nowhere to be had in my mom’s final days[. …] We received a sympathy card last week signed by only 1 name we recognized that had anything to do with our mom.”^b^

a For-profit hospice agency.

b Nonprofit hospice agency.

## Key Points

### Question

What are the most common criticisms found in negative (ie, 1-star) online reviews of hospice agencies?

### Findings

In this qualitative study of 137 unique online reviews of 67 hospice agencies, 2 domains of criticisms emerged: actionable criticisms, which are variables hospice organizations can change and include the themes of expectations, communication, and quality of care, and unactionable criticisms, which are variables that require larger systematic changes and include the themes of misperceptions about the role of hospice and the meaning of a good death.

### Meaning

In this study, many criticisms could have been mitigated through enhanced communication between staff and patients’ families, friends, and caregivers during hospice admission to foster realistic expectations of hospice staff, hospice services, and death.
